# Metabolic tracing reveals IL-2 driven glutaminolysis and *de novo* pyrimidine synthesis in human natural killer cells

**DOI:** 10.1016/j.jbc.2026.111367

**Published:** 2026-03-12

**Authors:** Karen Slattery, Gearóid Conlon, Ethan Collins, Derek P. Nolan, Clair M. Gardiner, Geraldine M. O’Connor

**Affiliations:** 1 School of Biochemistry and Immunology, Trinity Biomedical Sciences Institute, Trinity College Dublin, Dublin, Ireland; 2 School of Medicine and Dentistry, University of Lancashire, Preston, Lancashire, UK

**Keywords:** bioenergetics, glutamine, immunometabolism, innate immunity, natural killer cells, pyrimidine synthesis

## Abstract

Natural Killer (NK) cells are innate lymphocytes that are key to intrinsic cancer immunosurveillance and an important target for cancer immunotherapy. Understanding fundamental human NK cell metabolism provides opportunities for optimizing NK cell therapies. Little is known about how glutamine, an important cell nutrient and carbon source, is utilized by human NK cells. To address this, we performed U^13^C-glutamine tracing experiments by Liquid Chromatography Mass Spectrometry and Gas Chromatography Mass Spectrometry analysis of human NK cells stimulated with IL-2 for 18 h to provide a global overview of glutamine usage by these cells. Our results show that glutamine is taken up by resting NK cells and that this increases further upon IL-2 stimulation. Metabolite labelling analysis identified that IL-2 stimulation results in greater conversion of glutamine to glutamate, allowing for anaplerotic flux into the TCA cycle. The fate of the glutamine-derived carbons diverged at oxaloacetate allowing both bioenergetic and biosynthetic outcomes - some carbons continued around the TCA cycle while others were exported, converted to aspartate and subsequently used for pyrimidine synthesis. Nucleotide synthesis by IL-2 activated NK cells was found to be essential for expression of the activation marker CD69. The data indicate that glutamine is a key nutrient taken up by human NK cells, and that IL-2 drives glutaminolysis. Subsequent glutamate is used to support the TCA cycle, generating energy and providing intermediates for *de novo* pyrimidine synthesis.

Natural Killer (NK) cells are innate lymphocytes capable of rapid effector function, independent of prior exposure. Functioning primarily through cytotoxicity and cytokine production, NK cells contribute to control of malignancies in humans (1). Unfortunately, as cancer progresses, NK cell control of malignancy is often limited by the immunosuppressive tumor microenvironment. As a result, many NK cell immunotherapy strategies including cytokine-based stimulation, antibody-based therapies, adoptive cell transfer, and chimeric antigen-receptor (CAR) NK cells (2, 3) seek to prepare therapeutic cells to work, despite the tumor microenvironment. Extensive data focused on T cells has highlighted the need to understand immune cell metabolism for optimal cancer immunotherapy; however, our knowledge of basic NK cell metabolism is rudimentary.

In their resting state, human NK cells utilize low levels of glycolysis and oxidative phosphorylation to meet their energy requirements (4). Upon stimulation, these cells undergo metabolic reprogramming to meet the energetic, proliferative, and biosynthetic needs of the activated cell. Stimulation with cytokines such as IL-2 increases the uptake of glucose (primarily in the CD56^bright^ subset of NK cells) and increases both glycolytic activity and oxidative phosphorylation. This is believed to be controlled, at least in part, by upregulation of mammalian target of rapamycin (mTORC1).

Immune cells are known to utilize a range of carbon sources in addition to glucose (5) including the important amino acid glutamine, which is found in high concentrations in plasma and tissue fluids (6). Uptake of glutamine into the cell can be mediated by a number of amino acid transporters including SLC1A5, SLC38A1, SLC38A2 (reviewed in (7)), while import into the mitochondrion is mediated by a variant of SLC1A5 (8). Glutamine can provide energy *via* the Krebs cycle and contribute to the biosynthesis of important biomolecules molecules including nucleotides, glutathione, hexosamines, and non-essential amino acids (9).

Glutamine usage has been better studied in T cells. In their resting state, T cells are dependent on β-oxidation of fatty acids and pyruvate oxidation by the Krebs cycle for their energy requirements. Upon activation, these cells undergo metabolic reprogramming resulting in increased usage of glycolysis, the pentose phosphate pathway and glutaminolysis. Although initial stages of T cell activation are independent of available glutamine, these changes as proliferation and cytokine production require glutamine. Uptake is facilitated by an upregulation of glutamine transporters SLC38A1 and SLC38A2 (7) while upregulation of SLC1A5 (10) and increased uptake of glutamine from the medium was found to support Th1 and Th17 differentiation *via* mTORC1 activation.

Very little is known about the relative importance of glutamine as a carbon source in human NK cells. In murine NK cells, glutamine does not fuel oxidative phosphorylation, with levels of oxygen consumption in cytokine (IL-12 + IL-2) -activated NK cells unaffected by the absence of glutamine in the growth medium, inhibitors of the initial step in glutaminolysis glutaminase or a general glutamine antagonist (11). Instead, glutamine was essential in maintaining expression of the transcription factor c-myc which controls the metabolic changes including increased glycolysis and oxidative phosphorylation after cytokine stimulation. To address the gaps in our understanding of glutamine usage by human NK cells, we combined proteomic analysis with metabolite tracing to reveal the usage and fate of glutamine in resting and activated NK cells.

## Results

### IL-2 stimulates glutamine uptake and usage

**Figure 1:**
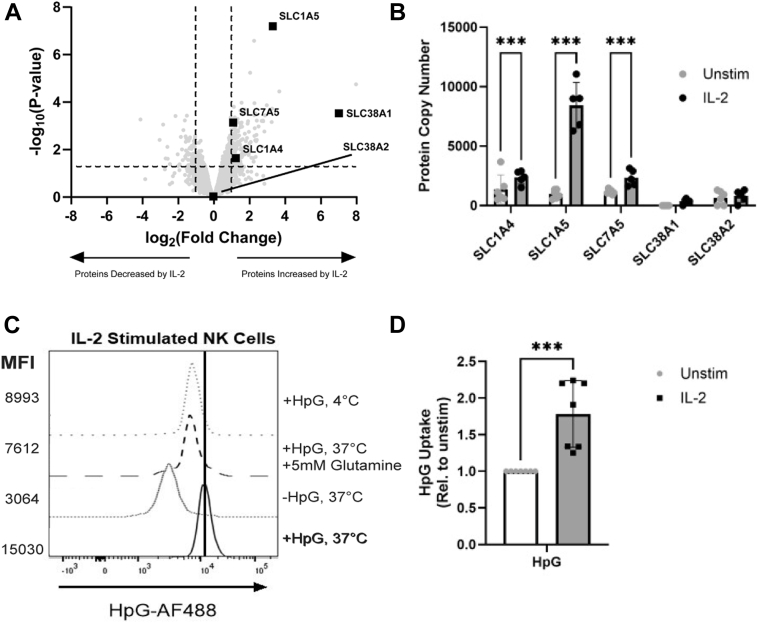
**Robust glutamine uptake by NK cells is further increased by IL-2**. *A*, purified human NK cells were stimulated with IL-2 (500 IU/ml) for 18 h and proteome analysis completed by mass spectroscopy. A volcano plot was generated from unpaired limma data (n = 5 IL-2 stimulated and n = 6 unstimulated) using protein copy numbers to highlight those proteins most highly up/down regulated by the treatment. *B*, expression of glutamine transporters in NK cells in resting or IL-2 treated conditions (mean ± SD, n = 5 [IL-2 stimulated] or n = 6 [unstimulated] independent biological replicates). Statistical analysis was performed using a two-way ANOVA with Sidak’s *post hoc* test (∗∗∗*p* < 0.001). *C*, PBMCs were cultured with HpG prior to fixation and subsequent copper click reaction with azide-AF488 to measure glutamine uptake (*D*) Uptake of glutamine by NK cells in resting and IL-2 treated conditions (mean ± SD, n = 7 independent biological replicates). Statistical analysis was performed using an unpaired, two tailed *t* test (∗∗∗*p* < 0.001). PBMC, peripheral blood mononuclear cell.

A proteomic analysis of IL-2 stimulated purified NK cells revealed an upregulation in glutamine transporters, with both SLC1A5 and SLC38A1 among the mostly highly upregulated proteins (Fig. 1*A*). Of the many glutamine receptors known, five were detected in our proteomic dataset: SLC1A4, SLC1A5, SLC7A5, SLC38A1 and SLC38A2 (Fig. 1*B*). SLC1A5 was the most expressed transporter and was dramatically upregulated by IL-2, suggesting that it may be the most important receptor for uptake of glutamine in human NK cells under these conditions. To explore the use of glutamine in NK cells, the capacity of peripheral blood NK cells to import glutamine was quantified by the uptake of the bioorthogonal amino acid homopropargylglycine (HPG) (12). Resting NK cells showed detectable HpG uptake, which was out-competed by unlabelled glutamine and absent at 4 °C (Fig. 1*C*). This uptake was significantly increased in NK cells stimulated for 18 h with IL-2 (Fig. 1*D*).

**Figure 2:**
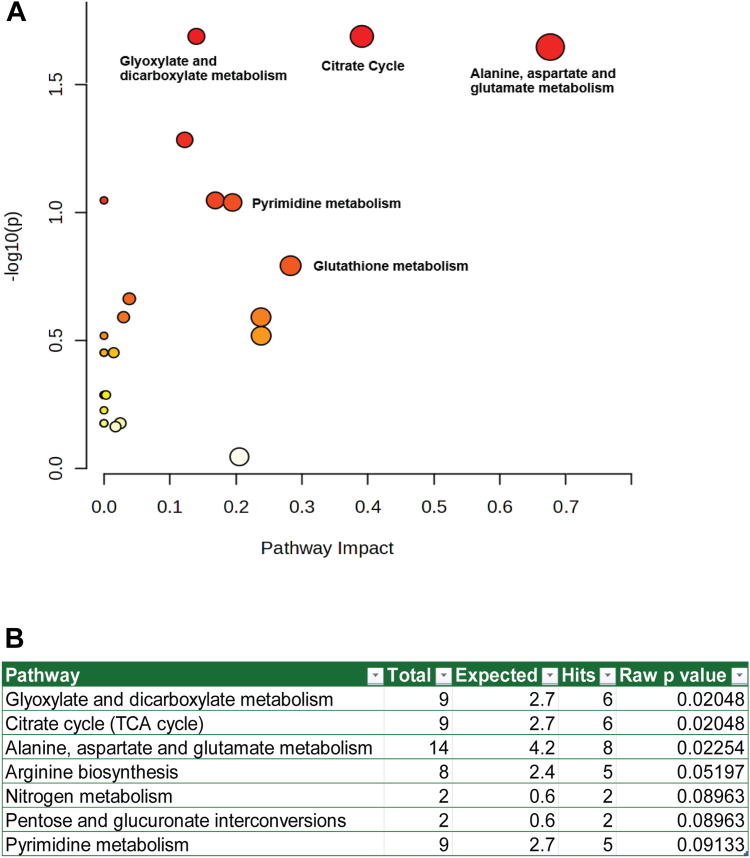
**NK cells utilize glutamine in a range of metabolic pathways NK cells were sorted by flow cytometry from the peripheral blood of healthy donors and stimulated in the presence of absence of IL-2 (500IU/ml) for 18h.** Glutamine in the medium was replaced with U-[^13^C]-glutamine to allow metabolic tracing of glutamine into various metabolites. Labelled metabolites identified by Liquid Chromatography Mass Spectrometry and GMCS underwent pathway analysis using MetaboAnalyst 6.0 to identified metabolic pathways associated with the glutamine-derived metabolites. *A*, dotplot highlighting the identified biological pathways based on the pathway impact (a function of both the centrality and pathway enrichment results), and the statistical significance of the finding. *B*, tabulated results for the pathway analysis showing the number of ^13^C labelled metabolites (“Hits”) compared to the total number of metabolites in the pathway (“Total”) and the number expected by chance (“Expected”). Table presents all results with a raw *p* values < 0.1; the adjusted *p* value did not show any statistically significant enrichment.

**Figure 3:**
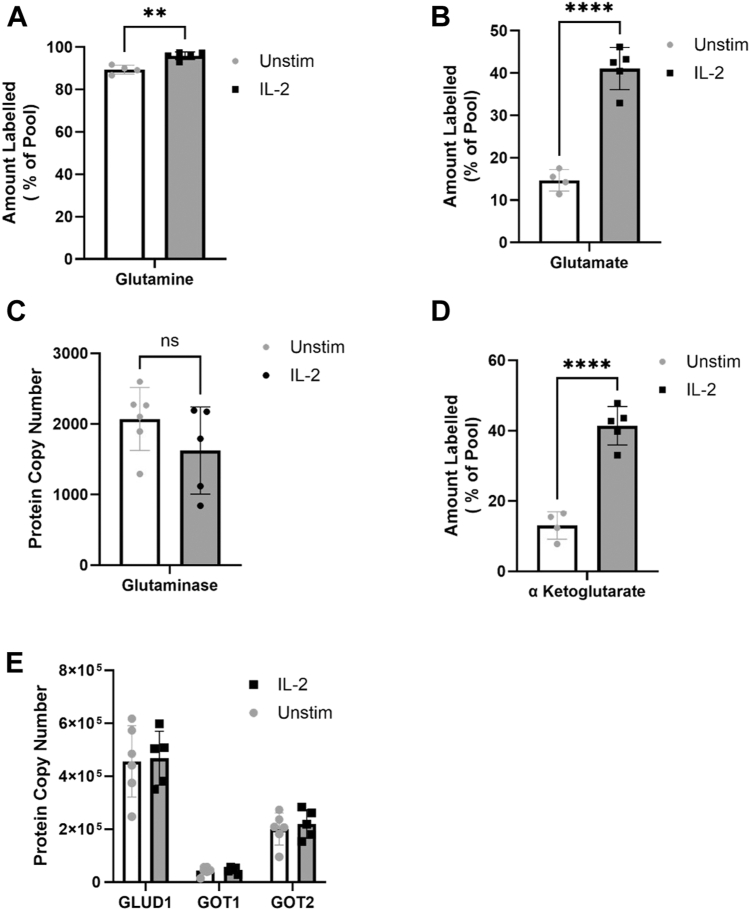
**Glutamine is used to generate the TCA intermediate α-ketoglutarate**. *A*, labelling of cellular glutamine following culture with U-[^13^C]-glutamine shown as proportion of total glutamine pool with labelled carbon (mean ± SD, n = 4 [unstimulated] or n = 5 (IL-2 stimulated] independent biological replicates). *B*, labelling of cellular glutamate following culture with U-[^13^C]-glutamine shown as proportion of total glutamate pool with labelled carbon (mean ± SD, n = 4 [unstimulated] or n = 5 IL-2 stimulated] independent biological replicates). *C*, proteomic analysis of expression of enzyme glutaminase (mean ± SD, n = 5 [IL-2 stimulated] or n = 6 [unstimulated] independent biological replicates). *D*, scatter plot shows labelling of cellular α-ketoglutarate following culture with U-[^13^C]-glutamine shown as proportion of total α-ketoglutarate pool with labelled carbon (mean ± SD, n = 4 [unstimulated] or n = 5 [IL-2 stimulated] independent biological replicates). *E*, NK cells were sorted by flow cytometry from the peripheral blood of healthy donors and stimulated in the presence of absence of IL-2 (500 IU/ml) for 18 h. Proteomic analysis of expression of enzymes glutamate dehydrogenase and mitochondrial glutamic-oxaloacetic transaminase (mean ± SD, n = 5 [IL-2 stimulated] or n = 6 [unstimulated] independent biological replicates). Statistical analysis was performed using an unpaired, two tailed *t* test (∗∗*p* < 0.01, ∗∗∗∗*p* < 0.0001).

Glutamine has many potential fates within a cell given that it plays a role in metabolism as a fuel source for ATP generation but also as a building block for proteins (as an amino acid) and nucleotides (as a nitrogen donor). To determine the metabolic fate of the large amounts of glutamine used by NK cells, we undertook metabolic tracing of U-[^13^C]-glutamine in purified NK cells cultured in the presence, or absence, of IL-2 for 18 h. Through a combination of both Liquid Chromatography Mass Spectrometry and Gas Chromatography Mass Spectrometry approaches, we identified a range of metabolites within the cell that derive from extracellular glutamine. Pathway enrichment analysis of these metabolites identified several biochemical pathways utilized by the NK cell – including glyoxylate and dicarboxylate metabolism; citrate cycle; alanine, aspartate and glutamate metabolism and pyrimidine metabolism (Fig. 2). Tracing analysis confirmed the glutamine uptake data with more than 80% of the cellular glutamine pool heavy-labelled after 18 h (Fig. 3*A*), even in the absence of cytokine stimulation. IL-2 resulted in a modest but significant increase in the amount of labelled glutamine in the cell. One key pathway of glutamine utilization highlighted by the pathway enrichment was glutamate and aspartate metabolism. After cellular uptake, glutaminolysis is the primary pathway to convert glutamine to glutamate in the mitochondria; glutamate is then used as a biochemical intermediate in immune cells for a variety of purposes. Transport of glutamine into the mitochondria is facilitated by a variant form of SLC1A5 (SLC1A5v); however, our proteomics did not distinguish this variant or indeed provide any subcellular localization of proteins; thus, the increase in SLC1A5 with IL-2 could in part be through increased expression of SLC1A5v. Evidence of glutaminolysis was provided by identification of heavy labelled glutamate in resting NK cells (approximately 12%, Fig. 3*B*). This proportion increased in the presence of IL-2 where it increased to represent ∼40% of the cellular glutamate pool, indicating a significant upregulation of glutaminolysis. The enzyme glutaminase, which hydrolyses glutamine to produce glutamate and ammonia, was readily detected in NK cells at a resting state, unchanged with IL-2 stimulation (Fig. 3*C*). Together, these data indicate that while human NK cells take up external glutamine basally, IL-2 ramps up the transporters required for glutaminolysis which in turn contributes a major source of glutamate within NK cells.

### Glutamine supports the production of the TCA cycle intermediate α-ketoglutarate

For entry into the TCA cycle, glutamate must first be converted to α-ketoglutarate in the mitochondria. Metabolic tracing detected labelling of α-ketoglutarate, with ∼12% of the cellular pool labelled at resting state, with a significant increase to ∼40% upon IL-2 stimulation (Fig. 3*D*), almost identical changes to those seen with glutamate labelling. There are a range of enzymes capable of converting glutamate to α-ketoglutarate, including the aminotransferases glutamic-oxaloacetic transaminase (GOT) and glutamic-pyruvate transaminase, and glutamate dehydrogenase 1 (GLUD1). Our proteomic analysis did not detect any expression of glutamic-pyruvate transaminase but both the cytosolic (GOT1) the mitochondrial glutamic-oxaloacetic transaminase (GOT2) variant of glutamic-oxaloacetic transaminase was present in resting NK cells and unaffected by IL-2 stimulation. GLUD1 was found at a higher level in NK cells and was similarly unaffected by the presence of IL-2 (Fig. 3*E*). Thus, NK cells activated with IL-2 metabolize glutamine to generate α-ketoglutarate.

### Glutamine serves as an anaplerotic substrate to generate TCA intermediates

**Figure 4:**
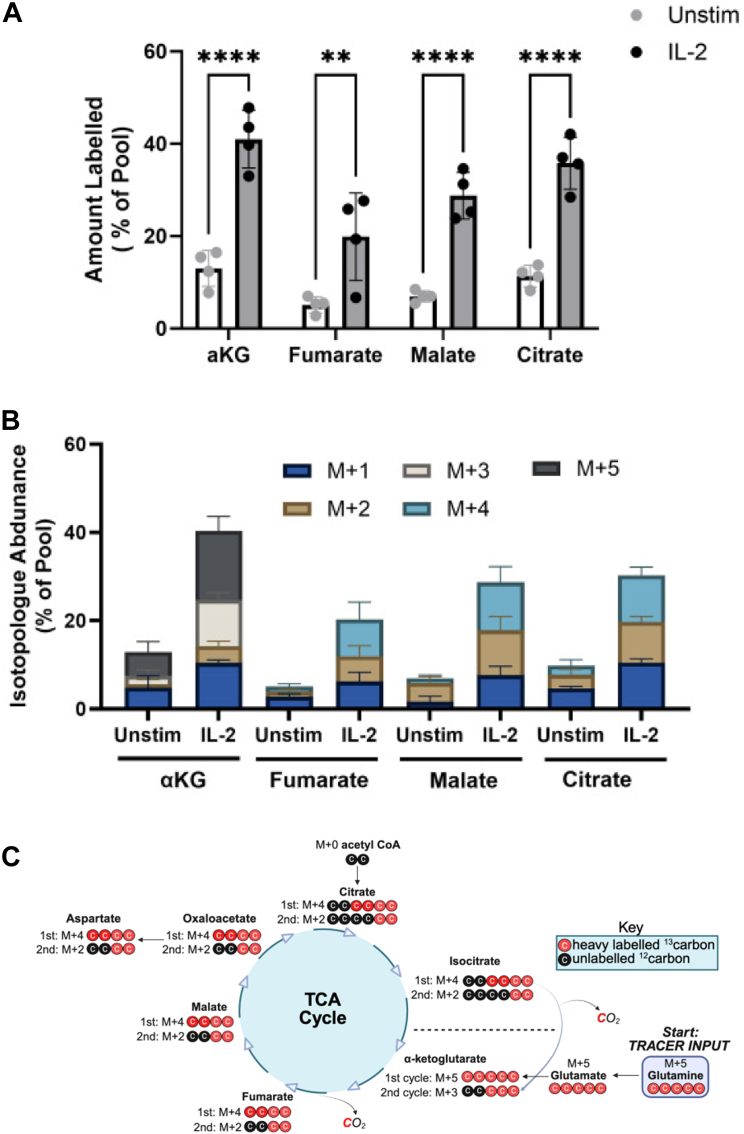
**Glutamine serves as an anaplerotic substrate to generate TCA intermediates**. *A*, labelling of cellular TCA intermediates following culture with U-[^13^C]-glutamine shown as proportion of total metabolite pool with labelled carbon (mean ± SD, n = 4 independent biological replicates). Statistical analysis was performed using a two-way ANOVA with Sidak’s *post hoc* test (∗∗*p* < 0.01, ∗∗∗∗*p* < 0.0001) *B*, MID analysis of TCA intermediates following culture with U-[^13^C]-glutamine shown as proportion of total metabolite pool, shown as proportion of total metabolite pool (mean ± SD, n = 4 independent biological replicates). *C*, schematic representation of entry of glutamine-derived labelled carbons into the TCA cycle after one and two cycles. Image created in https://BioRender.com. MID, mass isotopologue distribution.

Given that different TCA intermediates are siphoned off for specific purposes depending on the needs of the cell at a given point in time, ‘topping up’ or anaplerosis is required to maintain a functional TCA cycle. Our data indicate that glutamine serves to do this as metabolomic analysis of many of TCA intermediates paralleled the trend seen with glutamate and α-ketoglutarate. For fumarate, malate and citrate there was a small fraction of the pool labelled in the presence of U-[^13^C]-glutamine in resting NK cells which increased after stimulation with IL-2, rising from ∼5 to 12% to ∼20 to 40% dependent on the metabolite (Fig. 4*A*). Thus, labelled glutamine is being used to make other TCA intermediates. Isotopologue analysis, where individual TCA intermediates are assessed for the proportion of heavy labelled ^13^C-species, revealed the presence of a number of different labelled forms supporting that multiple cycles of the TCA occur. The dominant form of α-ketoglutarate was found to be the M+5 isotopologue (*i*.*e*.*,* five heavy labelled carbons), compatible with conversion from M+5 glutamine and M+5 glutamate. Loss of two labelled carbons *via* the action of α-ketoglutarate dehydrogenase and isocitrate dehydrogenase in turn explains the presence of M+3 and M+1 variants seen (Fig. 4*B*). Similarly, for later intermediates (fumarate, malate, citrate), the dominant form observed is M+4, in association with M+2. Together, these data show that IL-2 increases glutamine flux into TCA intermediates and isotopologue analysis is consistent with TCA cycling (Fig. 4*C*).

### Glutamine feeding of the TCA cycle allows production of aspartate

**Figure 5:**
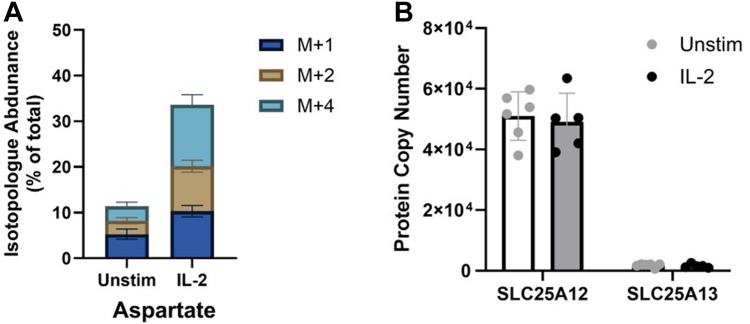
**IL-2 promotes production of aspartate from glutamine**. *A*, MID analysis of aspartate following culture with U-[^13^C]-glutamine shown as proportion of total metabolite pool, shown as proportion of total metabolite pool (mean ± SD, n = 4 independent biological replicates). *B*, proteomic analysis of expression of aspartate transporter (SLC25A12 and SLCA13) (mean ± SD, n = 5 [IL-2 stimulated] or n = 6 [unstimulated] independent biological replicates). MID, mass isotopologue distribution.

As mentioned, NK cells activated by cytokine need to adapt to changing metabolic needs. In addition to energy generation *via* cycling of the TCA, we found evidence in NK cells that glutamine feeding into the TCA supports the production of biosynthetic precursors, or cataplerosis. We focused on amino acids and in particular, aspartate which can be produced from oxaloacetate, a TCA intermediate downstream of malate (Fig. 4*C*). Analysis of the cellular pool of aspartate revealed that it is labelled after culture with U-[^13^C]-glutamine – with a small proportion of pool labelled at resting state which is increased by IL-2 (Fig. 5*A*), indicating that glutamine provides carbons for its synthesis. In line with the labelling pattern seen with other TCA intermediates, the dominant form of aspartate after glutamine tracing is M+4, with M+2 and M+1 isotopologues also readily detected. Conversion of oxaloacetate to aspartate is also catalysed by GOT2 (Fig. 3*E*) which has specific mitochondrial localization. Transport of this mitochondrial aspartate to the cytosol where it can be used in biosynthetic processes is dependent on the action of an aspartate-glutamate transporter. Of the two forms known, SLC25A13 (citrin) was detected at low levels in NK cells, but SLC25A12 (aralar) was found at much higher levels (Fig. 5*B*). These data demonstrate that glutamine is used to produce the amino acid aspartate in the mitochondria of activated NK cells and this is likely exported by SLC25A12 for use in biosynthesis reactions.

### Glutamine-derived aspartate is an important carbon source of pyrimidine production

**Figure 6:**
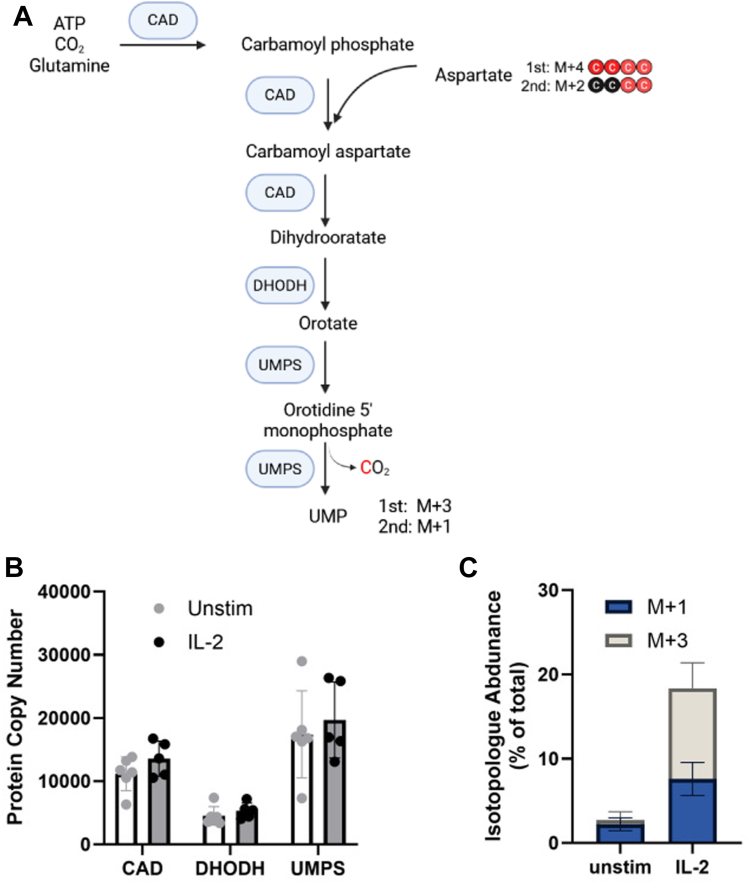
**IL-2 promotes *de novo* production of UMP**. *A*, overview of the metabolic pathways leading to the *de novo* production of pyrimidines and the enzymes involved. Predictions for patterns of carbon labelling based on aspartate labelling (after one or two cycles of U- [^13^C] glutamine-fed TCA cycle) are highlighted. Produced with BioRender. *B*, proteomic analysis of expression of pyrimidine biosynthetic enzymes including the trifunctional carbamoyl-phosphate synthetase 2, aspartate transcarbamylase, and dihydroorotase (CAD), DHODH, and uridine 5′-monophosphate synthase (mean ± SD, n = 5 [IL-2 stimulated] or n = 6 [unstimulated] independent biological replicates). *C*, MID analysis of UMP following culture with U-[^13^C]-glutamine shown as proportion of total metabolite pool, shown as proportion of total metabolite pool (mean ± SD, n = 4 independent biological replicates). MID, mass isotopologue distribution.

Pyrimidines (along with purines) are fundamental building blocks of both DNA and RNA nucleic acids within cells. *De novo* production of pyrimidines is dependent on a series of enzymatic reactions which use aspartate, derived from glutamine, to first generate uridine-monophosphate (UMP) for use directly, or to generate other pyrimidines. The trifunctional carbamoyl phosphate synthetase, aspartate transcarbamoyl and dihydrooratase (CAD) enzyme complex, which results in the production of dihydroorotate *via* the incorporation of aspartate to form the aromatic ring of pyrimidines (Fig. 6*A*). Proteomic analysis revealed that this enzyme is highly expressed in NK cells (Fig. 6*B*). The additional enzymes required for the formation of UMP, dihydroorotate dehydrogenase (DHODH), and uridine 5′-monophosphate synthase are also present in NK cells. Levels of these enzymes were not impacted by IL-2 stimulation (Fig. 6*B*). The end-product of these reactions, UMP was found to be labelled in tracing experiments with 20% label of the UMP pool indicating that is derived from glutamine. Although initially all four carbons from aspartate are incorporated into carbamoyl aspartate and in turn dihydroorotate, subsequent loss of CO_2_ results in three aspartate-derived carbons in UMP. In line with this, and the labelling observed of aspartate (Fig. 5*A*), we detected M+1 and M+3 labelled isotopologues of UMP, with levels increased by IL-2 (Fig. 6*C*). Thus, IL-2 stimulated NK cells engage in *de novo* pyrimidine synthesis using glutamine as a source of aspartate.

### Glutaminolysis and pyrimidine synthesis contribute to NK cell activation

**Figure 7:**
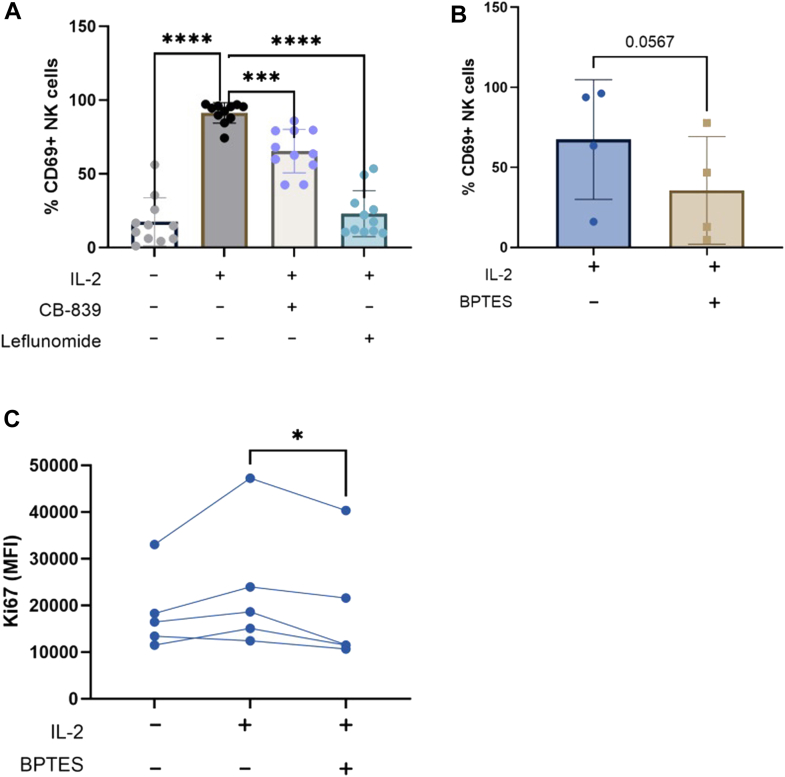
**Glutaminolysis and pyrimidine production contribute to NK cell activation**. *A*, PBMCs were cultured in the presence of IL-2 (500 IU/ml) for 18 h in the presence of the glutaminase inhibitor CB-839 or the inhibitor of DHODH, leflunomide. Expression of CD69 on the NK cell (CD56^+^ CD3^−^) population was measured by flow cytometry (mean ± SD, n = 11 independent biological replicates). Statistical analysis was performed using a one-way ANOVA with Dunnett’s multiple comparison *post hoc* test (∗∗∗*p* < 0.001, ∗∗∗∗*p* < 0.0001). *B*, PBMCs were cultured in the presence of IL-2 (500 IU/ml) for 18 h in the presence of the glutaminase inhibitor *N*,*N*'-[Thiobis(2,1-ethanediyl-1,3,4-thiadiazole-5,2-diyl)]bisbenzeneacetamide (BPTES). Expression of CD69 on the NK cell (CD56^+^CD3^−^) was measured by flow cytometry (mean ± SD, n = 4 independent biological replicates). Statistical analysis was performed using a paired, two tailed *t* test. *C*, PBMCs were cultured in the presence of IL-2 (500 IU/ml) for 18 h in the presence of the glutaminase inhibitor BPTES. Expression of Ki67 on the NK cell (CD56^+^CD3^−^) population was measured by flow cytometry (5 independent biological replicates), using total population MFI due to lack of clear discrimination between a positive and negative population. Statistical analysis was performed using a repeated measures one-way ANOVA with Tukey’s multiple comparison *post hoc* test (∗*p* < 0.05). PBMC, peripheral blood mononuclear cell.

To establish the functional importance of glutamine usage in NK cells, we examined NK cell activation *via* expression of the CD69 activation marker in the presence of inhibitors of the pathways identified above. CB-839, an inhibitor of glutaminase which prevents the conversation of glutamine to glutamate, was found to result in a partial but significant reduction in CD69 (Fig. 7*A*). The DHODH inhibitor leflunomide was found to have a profound effect on NK cell activation, with levels of CD69 in the presence of the inhibitor much reduced. Although DHODH will also have effects on oxidative phosphorylation which may also contribute to the impacts seen, this finding is consistent with a role of pyrimidine synthesis. To further strengthen our conclusion about the role of glutamine usage, we utilized a second glutaminase inhibitor *N*,*N*'-[Thiobis(2,1-ethanediyl-1,3,4-thiadiazole-5,2-diyl)]bisbenzeneacetamide (BPTES), which showed a decrease in IL-2 induced NK cells activation (Fig. 7*B*), although this did not reach statistical significance. This inhibitor also blunted the proliferative response of NK cells, reducing the upregulation of the proliferative marker Ki67 (Fig. 7*C*). Thus, perturbation of the glutamine-using pathways identified in NK cells inhibited NK cell IL-2-induced activation and proliferative response.

## Discussion

IL-2 is known to drive proliferation, upregulation of both glycolysis and oxidative phosphorylation, and enhance cytotoxic functions of human NK cells. Here, we demonstrate that NK cells increase glutamine uptake to help fuel these processes. Our data demonstrate that a key fate of imported glutamine in NK cells is entry into the TCA cycle. Labelling and tracing experiments demonstrate incorporation of glutamine-derived carbons into multiple TCA intermediates with isotopologue patterns consistent with active cycling. While one conventional output of the TCA is to fuel oxidative phosphorylation and generate ATP, it also uses biochemical intermediates to meet other metabolic needs of cells. Mechanistically, we show that glutamine-derived carbon in activated NK cells supports both anaplerosis and cataplerosis of the TCA. Specifically, isotopologue analysis showed that glutamine, through TCA intermediates, fueled aspartate synthesis which must be exported (most likely through SLC25A12) to the cytosol where tracing confirms contribution of carbons for *de novo* pyrimidine biosynthesis. Although our tracing specifically followed and identified the ‘carbon’ contribution of aspartate, glutamine would also be expected to contribute nitrogen to the pyrimidine synthesis (13), with pentose phosphate pathway-derived ribose also contributing (14). This metabolic demand parallels observations in proliferating T cells, where nucleotide synthesis is a major sink for glutamine-derived carbon and nitrogen (15) and extends the paradigm to innate lymphocytes.

Our data demonstrated that IL-2 drives significant glutamine uptake and glutaminolysis in human NK cells. Increased uptake of glutamine in these cells appears to be driven by increased expression of the transporter SLC1A5 rather than SLC38A1/2 which show only modest response to IL-2. SLC1A5 is also found to be upregulated in T cells in response to CD3/CD28 stimulation to drive increased glutamine uptake (16) and is important for both Th1 and Th17 cells (10). As mentioned above, glutamine cycles through the TCA and is siphoned off for aspartate to support *de novo* pyrimidine synthesis. It was interesting that none of the enzymes involved in glutaminolysis, α-ketoglutarate generation from glutamate (GLUD1, GOT1, GOT2) or indeed enzymes in the *de novo* pyrimidine synthesis pathway (CAD complex, DHODH, uridine 5′-monophosphate synthase) were upregulated by IL-2 in NK cells. This is despite 20% of the final UMP pool derived from labelled glutamine, and an increase of ∼12% to ∼40% increase in glutamate and α-ketoglutarate with IL-2 stimulation of NK cells. Thus, upregulation of key enzymes is not a major mechanism for upregulation these pathways. It remains a possibility that enzyme activity is regulated post-translationally. Indeed, for glutaminase it has been described that both phosphorylation and succinylation leads to increased activity (17, 18). Additionally, these NK cells may have reserve capacity to increase flux through the glutamine metabolic pathways. In the NK cell model NK-92 ectopic expression of a glutamine transporter (SLC1A1) alone was sufficient to increase not only glutamine levels but also a range of downstream metabolites including glutamate, aspartate and glutathione (19). Our data are clear that there is a huge uptake of glutamine, (>80% of glutamine labelling in unstimulated cells), suggesting that glutamine is key for basal homeostatic metabolic regulation. The dramatic upregulation of SLC1A5 with subsequent influx of cellular glutamine upon IL-2 activation, suggests that control of glutamine in human NK cells is through dynamic regulation of SLC1A5 expression levels.

We propose that increased *de novo* production of pyrimidines using glutamine-derived carbons is important for NK cell activation and proliferative response to IL-2. Our data did not identify any increase in the protein levels of the enzymes involved in this biochemical pathway, and thus it seems probable that stimulation leads to increased activity *via* other mechanism. One possible mechanism includes S6 kinase-dependent effects on the rate limiting step of pyrimidine synthesis (activity of the trifunctional CAD enzyme) (20, 21). NK cell stimulation with IL-2 has been described to lead to increased mTORC1 activity and in turn to S6 kinase activity (22), providing a potential mechanistic link between IL-2 stimulation and the biosynthetic pathways supporting proliferation seen.

Glutamine serves many functions in a cell - from an amino acid for protein synthesis, fuel for the TCA, through to regulation of redox homeostasis. Our data confirm its importance in human NK cells for *de novo* pyrimidine synthesis but also as a key fuel for the TCA. NK cells are generally considered to be metabolically plastic and are reported to be relatively insensitive to the loss of any individual fuel source in contrast to CD8+ T cells. Loss of glucose alone did not impact granzyme B level or interferon gamma production in response to IL-12/18 stimulation (23). Similarly, removal of glutamine as a fuel source had little functional impact and it was not until researchers targeted multiple fuel sources (glucose, glutamine, fatty acids) concurrently that there were appreciable impacts on IFN-γ production detected. Our findings here support this as inhibition of glutaminolysis in NK cells with CB-839 only partially inhibited CD69 expression, consistent with metabolic plasticity. There were similar functional findings in the murine system, where culture in glutamine-free conditions, while simultaneously inhibiting glycolysis and fatty acid oxidation did not blunt the IFN-γ response to IL-12/18 stimulation (24). However, murine NK cells have been reported to rely on glutamine primarily to maintain c-Myc expression rather than as a fuel source for oxidative phosphorylation (11). Our findings highlight that human NK cells use glutamine more extensively, both for fuelling the TCA cycle and for anabolic processes such as nucleotide biosynthesis. These data suggest important interspecies differences in NK cell metabolic wiring that may have implications for translation of preclinical findings into human settings.

Our data also have implications for NK cell-based immunotherapies for cancer. It is already known that chemotherapeutic agents such as 5-flourouracil and methotrexate that target pyrimidine synthesis pathways by different mechanisms are highly immunosuppressive through off-target effects on immune cells. Inhibition of DHOHD may have complex effects on the cell’s metabolism beyond its role in pyrimidine biosynthesis including impacts on oxidative phosphorylation (25). Specific DHODH inhibitors such as brequinar and leflunomide are being used in the clinic to treat autoimmune diseases such as multiple sclerosis, and are being explored as anti-cancer drugs (26). Along with a growing body of evidence that DHODH inhibitors damage T cells (27, 28), our results on human NK cells presented here suggest that such treatments could inadvertently suppress NK cell immunity and reduce therapeutic effect of NK-cell therapies. Careful analysis will be needed in terms of optimizing delivery of immune cell therapies to ensure their maximum therapeutic benefit.

Our findings also highlight the importance of understanding glutamine availability in the tumor microenvironment, which is often depleted by cancer cells and myeloid populations. While the impact of this on NK cell metabolic activities including nucleotide synthesis remain to be defined, it is likely that strategies to improve NK cell access to glutamine such as engineering expression of additional glutamine uptake receptors, or to bypass metabolic bottlenecks through provision of downstream metabolites (*e*.*g*. nucleoside supplementation), could enhance NK cell persistence and activity in tumors (29, 30). In summary, this first report of glutamine metabolic tracing in human NK cells provides new insights into the role of glutamine metabolism in human NK cells and demonstrates that IL-2 enhances glutamine uptake, glutaminolysis, and downstream biosynthetic processes such as *de novo* pyrimidine synthesis, which ultimately support NK cell activation.

## Experimental procedures

### Ethics

All blood samples for this study were collected in EDTA coated tubes. The analysis of healthy donor blood samples was approved by the Research Ethics Committee of School of Biochemistry and Immunology in Trinity College Dublin. All healthy donors for this study were consenting adults and study abides by the Declaration of Helsinki principles.

### Isolation of NK cells

Human peripheral blood mononuclear cells (PBMC) were isolated on the same day blood sample was drawn *via* density gradient centrifugation using Lymphoprep. Blood was combined with PBS at a ratio of 1:2, layered on top of lymphoprep (15 ml) and centrifuged for 30 min at 300*g* using a minimal brake. The buffy coat was extracted and washed in PBS (50 ml). PBMC were counted and stained with Live/Dead viability stain, CD56 (clone HCD56/NCAM16.2) and CD3 (SK7/UCHT1) by staining in FACS buffer (PBS + 2% fetal calf serum (FCS) for 20 min on ice. Live CD56^+^ CD3^−^ NK cells were purified by flow sorting using a BD FACSAria III Cell Sorter. Purity of sorted NK cells populations were confirmed by flow cytometry and was routinely >99% CD56^+^ CD3^−^, with no sample less than 95% CD56^+^ CD3^−^.

### Glutamine (L-homopropargylglycine, HpG) ‘click’ uptake assay

NK cells, in a PBMC preparation, were stained as above and resuspended in 100 μl HBSS. Samples were warmed in a cell incubator at 37 °C or chilled on ice at 4 °C (cold control) for 10 min. With the exception of the HpG negative control, 50 μl of L-Homopropargylglycine (HpG, 200 μM) was added to the cells. Glutamine was added to the competitive control at a final concentration of 5 mM. Samples were returned to the incubator or ice for 5 min. After this, cells were fixed with 50 μl of PFA (1%) in the dark for 30 min.

To perform the click chemistry, cells were first washed and resuspended in PBS at RT. PBMC were permeabilized in the dark for 20 min at RT in 200 μl permeabilisation buffer (1% BSA, 0.01% saponin). Cells were then centrifuged and washed with PBS and the plate dabbed on tissue to remove excess PBS. During this, a fresh preparation of “click-mix” was prepared as follows: copper sulfate (CuSO4, 1 mM), sodium ascorbate (NaAsc, 10 mM), tris-hydroxypropyltriazolylmethylamine (THPTA, 1 mM), aminoguanidine (10 mM), PBS and bioorthogonal fluorophore (AZDye 488 azide, 5 μM) were added together. Cells were resuspended in 30 μl of click-mix and incubated in the dark for 1h at RT. After washing in PBS at room temperature, cells were resuspended in cold buffer (PBS, 2% BSA) for flow cytometry.

### Culture of NK cells for proteomic analysis

Purified human NK cells from healthy adult donors (N = 6) were seeded at 1 x 10^6^ cells/ml in RPMI supplemented with 10% FCS and 1% penicillin/streptomycin and stimulated with IL-2 (500 IU/ml). Cells were then washed three times in HBSS (Gibco), aspirating off the remaining supernatant to ensure no BSA contamination and a dry pellet. Cell pellets and supernatants were then snap frozen using liquid nitrogen and kept at −80 °C until shipping for proteomic analysis at the University of Dundee. Proteomic sample preparation was performed by the technicians at the FingerPrints proteomic facility in the University of Dundee. Samples were processed using S-Trap Micro columns (Protifi), where proteins were reduced, alkylated, and digested for 24 h with 0.75 μg of trypsin per sample. A second digest step was performed for 6 h the following day. HiPPR detergent removal was performed to remove any remaining SDS from the digested peptides before further analysis was carried out. Digested peptides were run on an Orbitrap Astral mass spectrometer (Thermo Fisher Scientific) coupled to a Vanquish Neo UHPLC system (Thermo Fisher Scientific) with LC buffers comprising of 0.1% formic acid and 80% acetonitrile, 0.1% formic acid. Samples were run, and peptides were eluted from a PepMap RSLC C18 column (Thermo Fisher Scientific), and raw data were acquired in Data Independent Acquisition (DIA) mode. A scan cycle comprised a full MS scan with an m/z range of 380 to 940, resolution of 240,000, a custom Automatic Gain Control target of 500% and a maximum injection time of 5 ms. MS scans were followed by MS/MS DIA scans of dynamic window widths with an overlap of 0 m/z. DIA spectra were recorded with a scan range of 150 to 2000m/z, a custom Automatic Gain Control target of 500% and a maximum IT of 3 ms. Normalized collision energy was set to 25%. Data for MS scans were acquired in profile mode, with MS/MS DIA scan events being acquired in centroid mode. Data analysis was performed using Spectronaut v19 (https://biognosys.com/software/spectronaut; Biognosys) using directDIA. Samples were normalized using the global median. The Spectronaut output was used to calculate copy numbers using Perseus v1.6.12.0 (https://maxquant.org/perseus; MaxQuant) and the Proteomic Ruler plugin. Proteins quantified using one peptide and proteins undetected in >50% of samples were removed while one donor was excluded from paired analysis due to incomplete protein detection.

### Culture of NK cells for ^13^C-Glutamine tracing

2 x 10^6^ pure NK cells were seeded at 5 x 10^6^ cells/ml in glutamine-free RPMI medium supplemented with 10% dialyzed FCS, 1% penicillin/streptomycin and 2 mM ^13^C_5_-Glutamine (CK Isotopes Ltd), and stimulated with IL-2 (500 IU/ml) for 18 h at 37 °C, 5% CO_2_. Cells from independent biological replicates (n = 5 except for Liquid Chromatography Mass Spectrometry stimulated where n = 4) were then harvested, washed in ice cold saline solution twice and cell pellets lysed using ice cold 80% methanol prior to shipping to the Metabolomic Innovation Resource at McGill University for untargeted metabolomic analysis. Cellular metabolites were extracted and analyzed either by LC-MS/MS or GC-MS.

LC-MS: To follow incorporation of ^13^C-labeled glucose into glycolysis, pentose phosphate pathway, nucleotides and other central carbon metabolites, an LC- quadrupole time of flight ion pairing method was used. Six 1.4 mm ceramic beads were added to each sample. Samples were bead-beat for 2 m at 30 Hz and cleared by centrifugation. Clarified supernatant was transferred to a fresh pre-cooled tube and dried by vacuum centrifugation with sample temperature maintained at −4 °C (Labconco, Kansas City MO). Samples were resuspended in 50 μl UPLC grade water, clarified by centrifugation at 1 °C and transferred to LC/MS vials with 200 μl inserts. A volume of 5 μl was injected for stable isotope tracer analysis onto an Agilent 6530 quadrupole time of flight mass spectrometer. Chromatographic separation of metabolites was achieved by using a 1290 Infinity ultra-performance liquid chromatography system (Agilent Technologies). Eluent ionization was achieved using Agilent Jet stream electrospray ionization. The source-gas temperature and flow were set at 150 °C and 13 Lm^−1^, respectively. The nebulizer pressure was set at 45 psi and capillary voltage was set at 2000 V. Eluents were detected using negative ionization.

Chromatographic resolution of metabolites was achieved using a Zorbax Extend C18 column 1.8 μm, 2.1 × 150 mm^2^ with guard column 1.8 μm, 2.1 × 5 mm^2^ (Agilent Technologies). The gradient started at 100% mobile phase A (97% water, 3% methanol, 10 mM tributylamine, 15 mM acetic acid, 5 μM medronic acid) for 2.5 m, followed with a 5 m gradient to 20% mobile phase B (methanol, 10 mM tributylamine, 15 mM acetic acid, 5 μM medronic acid), a 5.5 m gradient to 45% mobile phase C (90% ACN) and a 7 m gradient to 99% B at a flow rate of 0.25 mL m^−1^. This was followed by a 4 m hold time at 100% mobile phase B. The column was restored by back-washing with 99% mobile phase C for 3 m at 0.25 mL m^−1^, followed by increase of the flow rate to 0.8 mL m^−1^ over 0.5 m and a 3.85 m hold, after which the flow rate was decreased to 0.6 mL m^−1^ over 0.15 m. The column was re-equilibrated at 100% A over 0.75 m, during which the flow rate was decreased to 0.4 mL m^−1^, and held for 7.65 m. One minute before the next injection, the flow was brought back to forward flow at 0.25 mL m^−1^. The column temperature was maintained at 35 °C. D-myrisitic acid was added as an internal standard and calibrations curves were run to ensure peak assignment. Authentic standards were reinjected periodically to ensure retention time stability. Stable isotope tracer data were analyzed, and matrix correction performed using Profinder software (https://www.agilent.com/en/product/software-informatics/mass-spectrometry-software/data-analysis/mass-profiler-professional-software/masshunter-profinder-mass-profiler-professional; Agilent Technologies). No corrections were made for potential ion suppression effects.

GC-MS: To better resolve TCA cycle intermediates and amino acids, the same samples were dried and subjected to GC-MS analysis as described previously (31). Briefly, methoxylamine HCl in pyridine was added to each sample followed by sylilation by N-(*tert*-butyldimethylsilyl)-N-methyltrifluoroacetamide. Data were analyzed using Mass Hunter Quant (Agilent technologies) and natural isotope abundance corrections applied as described previously (32).

The metabolite abundance was expressed relative to the internal standard and normalized to cell number. Metabolite spectra and retention times were verified by authentic standards for both GC-MS and LC-MS studies. Area under the curve for each metabolite and associated isotopes were analyzed and ensured to be below the saturation limit. Furthermore, in parallel to ^13^C-exposed samples, additional samples were prepared with natural abundance glucose or glutamine and treated the same as the ^13^C-labeled glucose and glutamine containing samples. These were used as control samples to ensure true ^13^C-incorportion as opposed to interfering ion(s).

Labelled metabolites were analysed *via* the MetaboAnalyst 6.0 online analysis software (https://dev.metaboanalyst.ca/) Pathway Analysis using a reference metabolome of all metabolites (labelled or unlabelled) detected.

### NK cell activation assay

PBMCs were isolated from the peripheral blood of healthy donors as described above and plated at 5 x 10^6^ cells/ml in RPMI V.1640 GlutaMAX medium supplemented with 10% FCS and 1% penicillin/streptomycin. Cells were stimulated with interleukin (IL)-2 (500 IU/ml) and incubated at 37 °C, 5% CO_2_ for 18 h. Inhibitors including CB-839 (100 nM), BPTES (*N*,*N*'-[Thiobis(2,1-ethanediyl-1,3,4-thiadiazole-5,2-diyl)]bisbenzeneacetamide, 20 μM) and leflunomide (100 μM) were added at time of stimulation as indicated.

Cells were washed in cold PBS and incubated with saturating concertation of antibodies to cell surface antigens (CD3, CD56, CD69, Ki67) in the presence of a LIVE/DEAD viability dye. Cells were washed, fixed and analysed *via* BD LSR Fortessa. Flow cytometric analysis was completed with FlowJo software (https://www.flowjo.com/flowjo/overview; Tree Star, Inc).

### Statistical analysis

Graphpad Prism was used for graphing and statistical analysis of data. Data are expressed as mean ± SD unless stated otherwise. Statistical significance of difference between two groups was evaluated *via* a two-tailed Student *t* test. Analysis of more than two groups was completed using a one-way ANOVA (one variable) followed by *post hoc* test (Dunnett’s multiple comparison test to compare all groups to control group or Tukey’s multiple comparison test to compare all groups to each other) or two-way ANOVA (two variables) followed by Sidak’s *post hoc* test.

## Data availability

The mass spectrometry proteomics data have been deposited to the ProteomeXchange Consortium via the PRIDE (33) partner repository with the dataset identifier PXD076428.

## Conflict of interest

The authors declare that they have no conflicts of interest with the contents of this article.
